# Cardiovascular–Endocrine–Metabolic Medicine: Proposing a New Clinical Sub-Specialty Amid the Cardiometabolic Pandemic

**DOI:** 10.3390/biom15030373

**Published:** 2025-03-05

**Authors:** Nikolaos Theodorakis, Maria Nikolaou, Andrew Krentz

**Affiliations:** 1 NT-CardioMetabolics, Clinic for Metabolism and Athletic Performance, 47 Tirteou Str., 17564 Palaio Faliro, Greece; 2 Department of Cardiology & Preventive Cardiology Outpatient Clinic, Amalia Fleming General Hospital, 14, 25th Martiou Str., 15127 Melissia, Greece; 3 School of Medicine, National and Kapodistrian University of Athens, 75 Mikras Asias, 11527 Athens, Greece; 4 School of Life Course & Population Health Sciences, King’s College London, London WC2R 2LS, UK; andrew.krentz@kcl.ac.uk

**Keywords:** cardiometabolic medicine, lifestyle medicine, cardiology, endocrinology, cardiovascular–renal–hepatic–metabolic syndrome, obesity, diabetes mellitus, heart failure, multiple hormonal deficiency syndrome

## Abstract

Cardiovascular–Renal–Hepatic–Metabolic diseases are on the rise worldwide, creating major challenges for patient care and clinical research. Although these conditions share common mechanisms and often respond to similar treatments—such as lifestyle changes and newer cardiometabolic drugs (e.g., SGLT2 inhibitors, GLP-1 receptor agonists)—clinical management remains divided among multiple specialties. Recently proposed curricula in Cardiometabolic Medicine and Preventive Cardiology reflect an effort to address this fragmentation. In addition, recent studies reveal that hormonal deficiencies may increase cardiovascular risk and worsen heart failure, with emerging data showing that correcting these imbalances can improve exercise capacity and possibly reduce major cardiac events. To overcome gaps in care, we propose a new sub-specialty: Cardiovascular–Endocrine–Metabolic Medicine. This approach unifies three main pillars: (1) Lifestyle medicine, emphasizing nutrition, physical activity, and smoking cessation; (2) the Integrated Medical Management of obesity, diabetes, hypertension, dyslipidemia, heart failure with preserved ejection fraction, early-stage kidney disease, metabolic-associated liver disease, and related conditions; and (3) hormonal therapies, focused on optimizing sex hormones and other endocrine pathways to benefit cardiometabolic health. By bridging cardiology, endocrinology, and metabolic medicine, this sub-specialty offers a more seamless framework for patient care, speeds up the adoption of new treatments, and sets the stage for innovative research—all critical steps in addressing the escalating cardiometabolic pandemic.

## 1. Introduction

Cardiovascular–Renal–Hepatic–Metabolic (CRHM) diseases have escalated to pandemic levels, propelled by obesity, insulin resistance, type 2 diabetes mellitus (T2DM), arterial hypertension, dyslipidemia, and hyperuricemia. These metabolic risk factors frequently co-occur and compound one another, increasing the likelihood of atherosclerotic cardiovascular disease (ASCVD), heart failure (HF), chronic kidney disease (CKD), and metabolic dysfunction-associated steatotic liver disease (MASLD). As these conditions progress, they further disrupt metabolic regulation, creating a vicious cycle of worsening outcomes [1]. Notably, HF with preserved ejection fraction (HFpEF) is now recognized as a distinct cardiometabolic entity, often referred to as the “non-alcoholic steatohepatitis (NASH) of the heart” [2].

A key link among CRHM diseases is the growing use of novel cardiometabolic drugs originally developed for glycemic control—namely sodium-glucose cotransporter-2 inhibitors (SGLT2is), glucagon-like peptide-1 receptor agonists (GLP-1RAs), and the dual gastric inhibitory peptide (GIP)/GLP-1RAs. Recent landmark studies have demonstrated their benefits across various cardiometabolic risk factors and established diseases, including obesity, HFpEF, coronary artery disease (CAD), CKD, MASLD, and obstructive sleep apnea (OSA), reshaping traditional specialty boundaries [3,4,5,6,7,8,9,10,11,12,13,14,15,16,17,18,19,20,21,22,23,24,25,26,27,28,29,30,31,32,33,34,35,36,37,38]. Meanwhile, lifestyle interventions remain vital in the treatment of these conditions, yielding multifaceted benefits in prognosis and quality of life.

In parallel, new evidence underscores the role of anabolic hormone deficiencies in CRHM disease risk and progression. Testosterone replacement in men with functional hypogonadotropic hypogonadism (FHH) has been shown to reduce the incidence of T2DM and may improve glycemic control and survival in men already diagnosed with T2DM [39,40]. The TRAVERSE trial further validated the cardiovascular safety of testosterone therapy (TTh) in men with FHH over two years [41], while data from the TOSCA registry indicate that more than 90% of HF patients have at least one anabolic hormone deficiency, correlating with poorer outcomes [42]. Meta-analyses also suggest that various hormonal replacement therapies can not only enhance cardiovascular function, symptoms, and exercise capacity but potentially improve major clinical endpoints [43,44,45,46,47,48]. This highlights the need to incorporate hormone-focused strategies into the broader management of HF and CRHM diseases.

**Figure 1:**
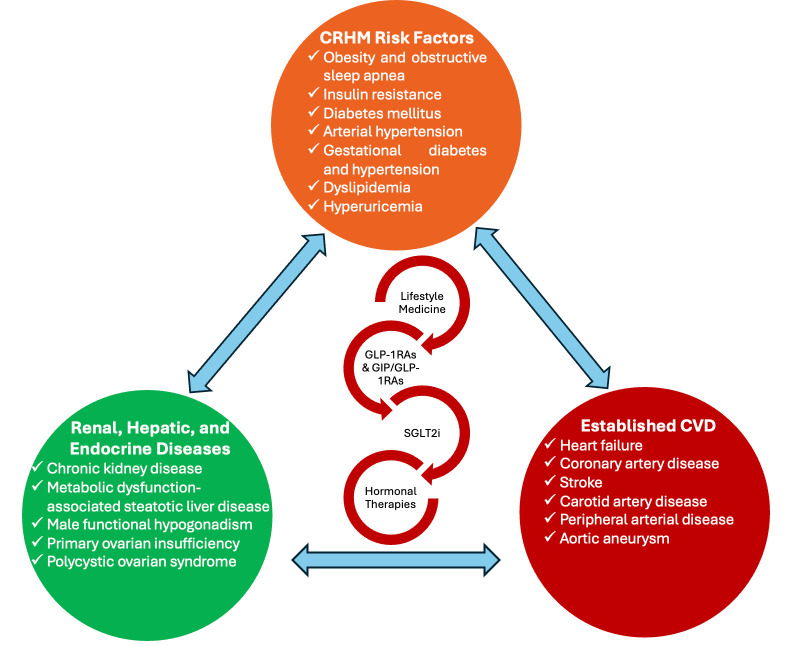
The interconnected relationships among cardiometabolic risk factors and established diseases and their overlapping management. The diagram illustrates the interconnected relationships among cardiometabolic risk factors, related hepatic, renal, and endocrine diseases, and established CVD. GLP-1RAs, including dual GIP/GLP-1RAs and SGLT2i, are emphasized for their roles in the integrated management of CRHM diseases, extending beyond their traditional diabetes indications. Lifestyle medicine serves as the foundation for prevention and management, complemented by anti-lipidemic medications (e.g., statins, anti-PCSK9) and antihypertensive agents, which address critical risk factors influencing the pathogenesis and progression of CRHM diseases. Additionally, hormonal therapies for conditions such as FHH, POI, and PCOS, as well as emerging evidence supporting their role in HFrEF, play an integral part in this comprehensive approach. Abbreviations: CAD (coronary artery disease); CKD (chronic kidney disease); CRHM (Cardiovascular–Renal–Hepatic–Metabolic syndrome); CVD (cardiovascular disease); FHH (functional hypogonadotropic hypogonadism); GIP (glucose-dependent insulinotropic polypeptide); GLP-1RAs (glucagon-like peptide-1 receptor agonists); HF (heart failure); HFrEF (heart failure with reduced ejection fraction); MASLD (metabolic-associated steatotic liver disease); OSA (obstructive sleep apnea); PAD (peripheral artery disease); PCOS (Polycystic Ovary Syndrome); PCSK9 (proprotein convertase subtilisin/kexin Type 9); POI (Primary Ovarian Insufficiency); SGLT2i (sodium-glucose cotransporter-2 inhibitors); T2DM (type 2 diabetes mellitus).

The interconnected relationships between cardiometabolic risk factors and established diseases, as well as their overlapping medical management and lifestyle interventions, are illustrated in Figure 1.

Despite the above advances, delivery of care for patients with CRHM diseases is recognized to be fragmented across clinical specialties such as cardiology, endocrinology, nephrology, hepatology, and internal medicine-diabetology. This fragmented approach often leads to redundant diagnostic tests, conflicting medical advice, medication errors, and adverse drug–drug interactions that ultimately result in suboptimal outcomes [49,50,51,52,53]. In recognition of this challenge, new clinical sub-specialties have been proposed to address the gaps in care. For instance, Preventive Cardiology has emerged to emphasize the risk reduction and prevention of cardiovascular events, mostly in primary prevention contexts but also in secondary contexts [54,55]. Furthermore, Cardiometabolic Medicine has been conceptualized to integrate the management of cardiometabolic diseases [56,57].

In this paper, we propose a new sub-specialty—Cardiovascular–Endocrine–Metabolic Medicine—that expands and elaborates upon existing proposals such as Cardiometabolic Medicine and Preventive Cardiology. This sub-specialty not only integrates cardiovascular prevention and the management of CRHM diseases but also incorporates hormonal therapies as an important and still relatively underappreciated focus of research and future clinical practice. Our proposal is anchored on three interconnected pillars:

■ Lifestyle Medicine: Incorporating evidence-based strategies, this pillar focuses on the integration of nutritional, exercise, and behavioral interventions to prevent and manage CRHM diseases. Inspired by principles observed in longevity-focused so-called Blue Zones and the pillars of lifestyle medicine described in our previous manuscript, this approach seeks to modify key lifestyle factors such as diet, physical activity, sleep, stress management, and smoking–alcohol cessation to enhance both health span and life span [58].
■ Integrated Medical Management of CRHM Diseases: This pillar focuses on the optimization of the medical management of the cardiometabolic spectrum, including obesity, insulin resistance, T2DM, arterial hypertension, dyslipidemia, cardiometabolic HFpEF, early-stage CKD, MASLD, OSA, FHH, and polycystic ovarian syndrome (PCOS). Recognizing the shared pathophysiology and overlapping therapeutic strategies, this framework promotes an integrated approach to diagnosis, prevention, and treatment.
■ Hormonal Therapies for CRHM: This research involves hormonal therapies for HF, integration of TTh for men with FHH and CRHM diseases, and optimized care for women with premature ovarian insufficiency (POI).

**Figure 2:**
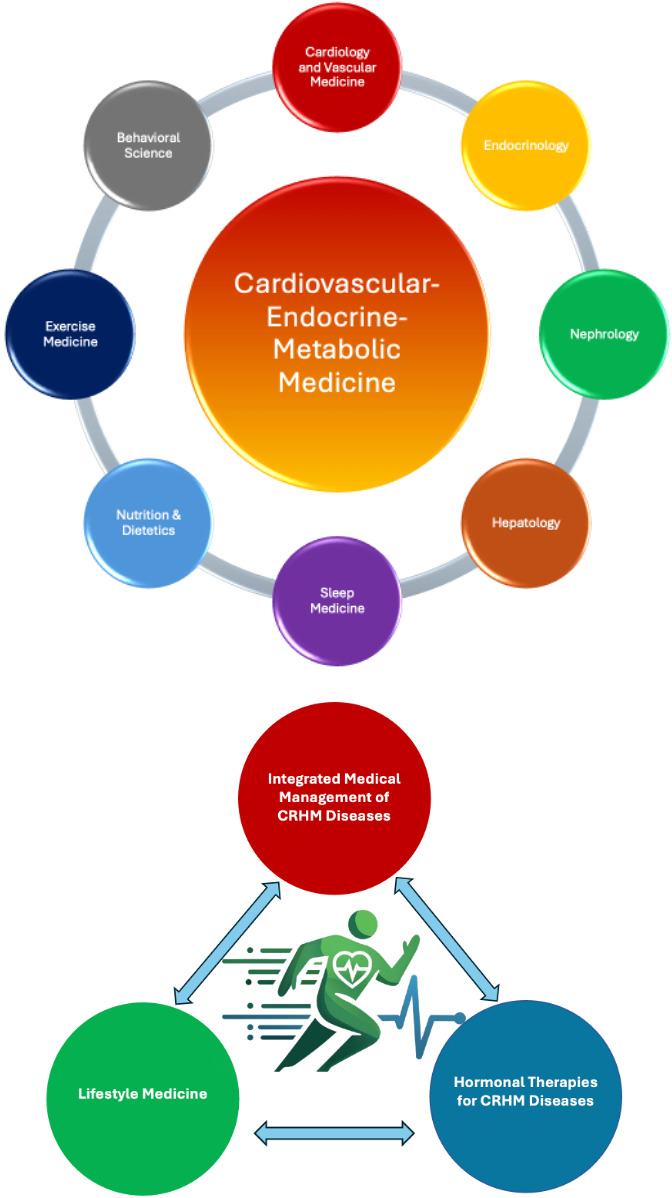
The three pillars and the related disciplines around the sub-specialty of Cardiovascular–Endocrine–Metabolic Medicine.

These three pillars and the related disciplines around the sub-specialty of Cardiovascular–Endocrine–Metabolic Medicine are illustrated in Figure 2. This framework not only integrates advances in cardiology, endocrinology, nephrology, hepatology, clinical nutrition, and exercise medicine but also bridges critical gaps in interdisciplinary training. By proposing structured curricula and clearly defining core competencies, Cardiovascular–Endocrine–Metabolic Medicine aims to equip clinicians with the tools needed to deliver integrated, patient-centered care, accelerating the implementation of groundbreaking therapies.

## 2. The Cardiometabolic Pandemic

CVD is the leading cause of mortality worldwide. Over the past few decades, significant advancements in prevention and management strategies have led to notable declines in CVD mortality rates [59]. For example, in Greece, the age-standardized mortality rate (ASMR) for CVD has decreased by an impressive 42.7% over the past 20 years [59]. However, this downward trend has plateaued and is now reversing in some regions, including Greece and the United States. In the United States, the ASMR for CVD declined by 8.9% between 2010 and 2019 (from 456.6 to 416.0 per 100,000) but subsequently increased by 9.3% from 2019 to 2022, reaching 454.5 per 100,000—essentially returning to its 2010 level [60]. In the UK, the incidence of CVD declined between 2000 and 2019, largely due to reductions in ischemic heart disease and stroke rates [61]. Improvements in rates of coronary heart disease were confined to people aged >60 years. Moreover, there was a rise in cardiac arrhythmias, valve disease, and thromboembolic events over this period [61]. These trends reflect a broader global phenomenon, highlighting the urgent need for strategies to address the evolving burden of CVD.

The rising prevalence of metabolic disorders, notably obesity and T2DM, is hypothesized to be driving the resurgence of CVD mortality. Both T2DM and obesity are leading risk factors for ASCVD, HF, CKD, and MASLD. According to the World Health Organization, the global prevalence of adult obesity has more than doubled between 1990 and 2022, while adolescent obesity has quadrupled. As of 2022, 43% of adults were classified as overweight, and 16% were considered obese [62]. Furthermore, according to 2019 Eurostat data, 16.1% of the European population were recorded as obese (16.4% among males, 15.9% among females), and 52.7% were overweight (60.2% among males, 45.7% among females) [63]. Similarly, the global prevalence of T2DM has surged by over 50%, rising from below 4000 per 100,000 in 1990 to approximately 6000 per 100,000 in 2017, with projections exceeding 7000 per 100,000 by 2040. In Western Europe, T2DM prevalence has increased by 50%, climbing from below 6000 per 100,000 in 1990 to approximately 8500 per 100,000 in 2017 [64]. Compounding these trends, HFpEF is now recognized as a distinct cardiometabolic disease, with its development primarily driven by aging, obesity, insulin resistance, T2DM, arterial hypertension, and systemic inflammation. HFpEF accounts for approximately 65% of all HF cases and has a global prevalence of 1.4%, with incidence rates continuing to rise [65].

## 3. First Pillar: Lifestyle Medicine

Lifestyle medicine is fundamental in managing CRHM diseases, focusing on modifying behavioral risk factors to prevent and treat the totality of CRHM diseases. Evidence-based interventions—including weight management, physical activity, nutrition, and smoking cessation—significantly improve long-term outcomes in these disorders. Patient education is critical in this process, enhancing adherence to treatment and improving prognosis, as demonstrated by a second-order meta-analysis [66].

### 3.1. Nutrition

Diets rich in whole grains, fruits, vegetables, low in saturated and trans fats, and rich in monounsaturated and polyunsaturated fats found in olive oil, nuts, and fatty fish—such as the Mediterranean or DASH (Dietary Approaches to Stop Hypertension) diets—are effective in lowering BP, lowering low-density lipoprotein cholesterol (LDL-C) and triglyceride levels by up to 10%, increasing high-density lipoprotein cholesterol (HDL-C) by up to 10%, improving glycemic control, and reducing cardiovascular risk [23,67,68,69]. A high intake of refined carbohydrates and added sugars, such as fructose and sucrose, worsens lipid profiles, increases triglycerides, reduces HDL-C, and impairs glycemic control. Reducing the consumption of these simple sugars and emphasizing complex carbohydrates and soluble fiber—found in whole grains, oats, barley, and legumes—can improve insulin sensitivity and lipid profiles and reduce cardiovascular risk [23,67,68,69].

Excessive dietary sodium intake is positively correlated with elevated BP and an increased risk of cardiovascular events. Reducing sodium intake to less than 2 g per day (approximately 5 g of salt) can result in significant reductions in systolic BP, with decreases of up to 5–6 mmHg in hypertensive patients. Notably, pooled data from salt-reducing trials have shown that reducing salt by 2.5 g/day is associated with a 20% decrease in the risk of cardiovascular events [70]. Concurrently, increasing dietary potassium intake—through the consumption of fruits, vegetables, and legumes—can further lower BP and mitigate the adverse effects of high sodium intake. An increase in potassium intake by 0.5–1.0 g/day may be considered to achieve a Na^+^/K^+^ ratio of 1.5–2.0, as proposed by the 2024 ESC guidelines with a class IIa recommendation. However, caution is warranted in patients with CKD and/or those taking potassium-sparing medications [23,67,68,69]. Energy drinks containing sugar and sodium and sugar-sweetened beverages should be avoided due to their potential to elevate BP and contribute to weight gain [23,67,68,69].

### 3.2. Physical Activity and Exercise

Physical activity and exercise are critical components of lifestyle medicine. Physical activity refers to any bodily movement produced by skeletal muscles resulting in energy expenditure, while exercise is a subset of physical activity that is planned, structured, and repetitive and aims to improve or maintain physical fitness [23,67,68,69].

Regular physical activity, including both aerobic exercise and resistance training, of sufficient intensity and duration offers significant benefits in managing hypertension and other cardiometabolic risk factors. Aerobic exercise, such as brisk walking or cycling for at least 150 min per week, can reduce systolic BP by approximately 7–8 mmHg and diastolic BP by 4–5 mmHg [71,72]. Dynamic resistance training and isometric exercises also contribute to BP reduction and improve vascular health [23,67,68,69].

Furthermore, engaging in regular physical activity favorably modifies lipid profiles by decreasing LDL-C levels by up to 5% and increasing HDL-C levels by more than 10%. Additionally, exercise interventions have been shown to reduce glycated hemoglobin (HbA1c) levels by approximately 0.6% in patients with T2DM [73]. Combining aerobic exercise with resistance training yields greater improvements in glycemic control compared to either modality alone, which can be attributed to enhanced insulin sensitivity and increased glucose uptake by skeletal muscles via GLUT4 [23,67,68,69].

Higher levels of total physical activity are associated with a lower risk of cardiovascular mortality and all-cause mortality. In a prospective cohort and meta-analysis, individuals with high total physical activity had a hazard ratio of 0.60 (95% CI, 0.49–0.73) for all-cause mortality compared to those with low physical activity levels [74].

Exercise prescriptions should be individualized, considering the patient’s age, comorbidities, physical fitness level, and personal preferences. Consistency is key to maintaining BP reductions and other health benefits. The World Health Organization recommends that adults engage in at least 150 min per week of moderate-intensity aerobic physical activity or at least 75 min per week of vigorous-intensity aerobic activity or an equivalent combination. Muscle-strengthening activities involving major muscle groups should be performed on two or more days per week. Patients should be encouraged to increase their daily physical activity by incorporating more steps, using stairs instead of elevators, walking or cycling for transportation, and minimizing sedentary behavior [23,67,68,69].

### 3.3. Weight Management

Obesity, particularly central adiposity, is strongly associated with insulin resistance, T2DM, arterial hypertension, dyslipidemia, and increased cardiovascular risk. Weight reduction improves these risk factors and reduces mortality. A modest weight loss of 5% can lead to significant health benefits, including improvements in glycemic control and reductions in systolic and diastolic BP by approximately 4.4 mmHg and 3.6 mmHg, respectively [75]. Weight loss of 5–10% can decrease LDL-C and triglyceride levels by up to 10% and increase HDL-C levels by up to 10%. Maintaining a normal body mass index (BMI) between 20 and 25 kg/m² and a waist circumference of less than 94 cm in men and less than 80 cm in women is crucial to ensuring cardiovascular risk. Lower targets for these measures of adiposity, with their well-recognized caveats, may be optimal for some populations [23,67,68,69].

Lifestyle interventions for weight management primarily include adopting a hypocaloric diet and increasing physical activity. Combining dietary calorie restriction with regular exercise is more effective for weight loss than either intervention alone. Various dietary approaches—such as low-calorie diets, low-carbohydrate diets, and low-fat diets—have been effective when adherence is maintained. While intermittent fasting has gained popularity, recent studies suggest that certain patterns of intermittent fasting may not confer additional cardiovascular benefits and may even pose risks in some populations; however, evidence is mixed, and more research is needed [76].

### 3.4. Smoking, Alcohol and Caffeine

Alcohol consumption has a dose-dependent relationship with BP and cardiovascular risk. Even moderate alcohol intake (up to one drink per day for women and up to two drinks per day for men) can increase BP, worsen glycemic control, and elevate triglyceride levels. Limiting alcohol intake is crucial in managing CRHM diseases. Moderate coffee consumption (3–4 cups per day) is generally considered safe and may confer cardiovascular benefits due to its antioxidant properties. However, excessive caffeine intake can cause transient increases in BP and arrhythmias in susceptible individuals [23,67,68,69].

Smoking is a major modifiable risk factor for CVD, contributing to endothelial dysfunction, inflammation, oxidative stress, and thrombogenesis. It acutely raises BP and heart rate, exacerbates insulin resistance, decreases HDL-C levels, and increases LDL-C oxidation. Smoking cessation is paramount in reducing cardiovascular risk. Comprehensive cessation programs combining behavioral counseling with pharmacotherapy (e.g., nicotine replacement therapy, varenicline, bupropion) are more effective than minimal interventions. GLP-1RAs are being evaluated as a potential new option to aid smoking cessation. Clinicians should actively engage patients in smoking cessation efforts and provide ongoing support [23,67,68,69].

### 3.5. Integrating Lifestyle Medicine into Cardiovascular–Endocrine–Metabolic Medicine

Integrating lifestyle medicine into *Cardiovascular*–*Endocrine*–*Metabolic Medicine* necessitates an interdisciplinary, patient-centered approach. Clinicians should develop competencies in nutritional counseling, exercise prescription, and behavior change techniques to effectively design and implement comprehensive lifestyle intervention programs. This holistic approach can address the multifactorial and interconnected nature of CRHM diseases, targeting multiple risk factors simultaneously [23,67,68,69].

By establishing lifestyle medicine as a fundamental pillar, the proposed sub-specialty aims to bridge gaps in care delivery, slow disease progression, and enhance long-term clinical outcomes. Continuous patient education, motivational support, and regular monitoring are critical components of successful lifestyle interventions. Incorporating technological advancements such as telemedicine, wearable devices, and mobile health applications can further facilitate patient engagement and adherence to lifestyle modifications [23,67,68,69].

**Figure 3:**
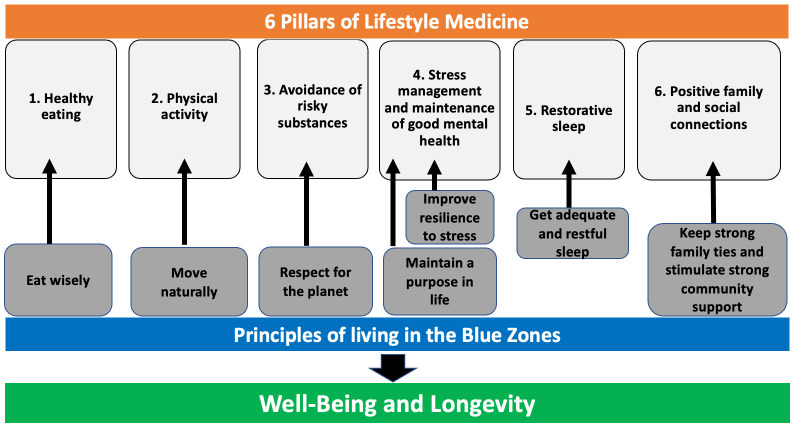
Mapping the six pillars of lifestyle medicine to the principles of living in the Blue Zones. This diagram outlines the “6 Pillars of Lifestyle Medicine”, which emphasize a holistic approach to achieving well-being and longevity, incorporating principles from the “Blue Zones”. Modified and used with permission from Kreouzi et al. (2022) [58].

Lifestyle medicine interventions have proven to be crucial in promoting healthy aging and longevity. As described in our previous manuscript, individuals living in Blue Zones—regions known for their extreme longevity—naturally incorporate habits into their everyday lives that align with the recommendations of the American Society of Lifestyle Medicine, as illustrated in Figure 3 [58].

## 4. Second Pillar: Integrated Medical Management of Cardiovascular–Renal–Hepatic–Metabolic Diseases

*Cardiovascular*–*Endocrine*–*Metabolic Medicine* has a strong focus on integrating the medical management of CRHM diseases. This approach encompasses the optimization of the medical management of obesity, insulin resistance, T2DM, arterial hypertension, dyslipidemia, hyperuricemia, cardiometabolic HFpEF, early-stage CKD, MASLD, OSA, and PCOS. These conditions have an interconnected pathophysiology and often have overlapping medical management.

### 4.1. Obesity, T2DM, Arterial Hypertension, and Dyslipidemia

The contribution of cardiologists to diabetes care has been well documented, even before the widespread adoption of glucose-lowering medications with proven cardiovascular benefits in HF and ASCVD. In the United States in 2016, cardiologists were the most frequently involved specialists in diabetes care (*n* = 22,848), followed by endocrinologists (*n* = 7793) and nephrologists (*n* = 7504). Additionally, at a tertiary care center, the ratio of cardiologist-to-endocrinologist outpatient encounters among patients with diabetes was approximately 3:1. This ratio was even higher—around 5:1—for patients with comorbid CVD [77]. Currently, obesity and T2DM are primarily managed by endocrinologists and internists–diabetologists, even though their most serious complications—cardiovascular and renal—fall within the expertise of cardiologists and nephrologists. The lack of integrated care often complicates management, especially for patients with established ASCVD, HF, or CKD. While collaboration between cardiologists, endocrinologists, and nephrologists is clearly important for these patients, it often leads to fragmented care, medication errors, conflicting advice, the duplication of laboratory tests, patient confusion, and ultimately suboptimal clinical outcomes [49,50,51,52,53]. In contrast, arterial hypertension—a systemic disease like T2DM that promotes vascular complications—is primarily managed by cardiologists and primary care physicians. Given that both T2DM and arterial hypertension are key risk factors for the same complications—ASCVD, HF, CKD, and retinopathy—it raises the question of why their management is divided between endocrinology and cardiology rather than being unified under a single expert with comprehensive knowledge of both fields. The management of arterial hypertension is further complicated by the involvement of multiple specialties in specific contexts. Endocrinologists often play a role in cases of secondary hypertension, as several common causes are endocrine in origin, including hypothyroidism, hyperthyroidism, primary hyperaldosteronism, Cushing’s syndrome, primary hyperparathyroidism, and pheochromocytoma [78]. Similarly, nephrologists play a crucial role in managing hypertension, particularly for patients with primary arterial hypertension complicated by CKD, as well as those with nephrogenic causes of secondary hypertension, including CKD of any origin, glomerular diseases, and scleroderma renal crisis [79].

Dyslipidemia—which is a condition with complex bidirectional associations with other CRHM diseases—is managed across multiple specialties, including cardiology, endocrinology, internal medicine, and primary care. This underscores the persistent fragmentation of care in Cardiometabolic Medicine, particularly in the era of specialized treatments for dyslipidemia. These treatments include proprotein convertase subtilisin/kexin type 9 (PCSK9) inhibitors and an expanding body of research on small interfering RNA (siRNA) therapies targeting lipoprotein(a) [68,80,81].

The management of obesity is central to cardiometabolic care. Recent advances in obesity management have demonstrated significant potential for weight reduction through the agonism of incretin receptors, as highlighted by several landmark trials. The STEP 1 trial evaluated semaglutide 2.4 mg weekly for 68 weeks in 1961 adults without diabetes, achieving an average weight loss of −14.9% (−15.3 kg) compared to −2.4% (−2.6 kg) with a placebo (treatment difference: −12.4% [−12.7 kg], 95% CI: −13.4 to −11.5, *p* < 0.001) [19]. Similarly, the STEP 2 trial in 1210 adults with T2DM reported a weight loss of −9.6% (−9.6 kg) with semaglutide at 2.4 mg compared to −3.4% (−3.6 kg) with a placebo (difference: −6.2% [−6.0 kg], 95% CI: −7.3 to −5.2, *p* < 0.0001) over 68 weeks [20].

The SURMOUNT-1 trial assessed tirzepatide (a dual glucose-dependent insulinotropic polypeptide (GIP)/GLP-1 receptor agonist) in 2539 adults over 72 weeks, demonstrating dose-dependent weight losses of −15.0% (−15.7 kg), −19.5% (−20.9 kg), and −20.9% (−22.5 kg) with tirzepatide at 5 mg, 10 mg, and 15 mg, respectively, versus −3.1% (−3.2 kg) with a placebo (*p* < 0.001 for all comparisons) [31]. The SURMOUNT-2 trial extended these findings in patients with T2DM, where tirzepatide at 15 mg achieved −13.8% (−13.8 kg) weight loss compared to −3.3% (−3.6 kg) with a placebo (*p* < 0.001) [32]. In the extended analysis of SURMOUNT-1 participants with prediabetes, three years of tirzepatide intake at 15 mg reduced the risk of progression to T2DM by 93% (hazard ratio: 0.07, 95% CI: 0.0 to 0.1, *p* < 0.001), corresponding to an absolute risk reduction of 12.4% (13.7% incidence with placebo vs. 1.3% with tirzepatide). This significant reduction in diabetes risk was accompanied by substantial and sustained weight reductions, with participants achieving an average weight loss of −19.7% (−21.3 kg) from baseline at 176 weeks compared to −1.3% (−1.4 kg) with a placebo. After the 17-week off-treatment period, the tirzepatide group maintained a weight reduction of −17.3% (−18.7 kg) relative to baseline [33]. Notably, the phase 2 trial of retatrutide, a novel triple GIP/GLP-1/glucagon receptor agonist, showed substantial efficacy, with the 12 mg dose achieving −24.2% (−23.6 kg) at 48 weeks compared to −2.1% (−2.1 kg) with a placebo (*p* < 0.001, 95% CI: −26.1 to −22.3) [34].

### 4.2. SGLT2i and GLP-1RAs: A Wide Repertoire of Indications

The management of HF, CAD, and CKD increasingly incorporates anti-diabetic medications. For HF, SGLT2 inhibitors have become a standard guideline-directed medical therapy across the entire spectrum of the left ventricular ejection fraction, regardless of diabetes status, according to recent landscape-event-driven trials (EMPEROR-Preserved, EMPEROR-Reduced, DELIVER, DAPA-HF) [3,4,5,6,7,8,9,10].

The use of anti-diabetic medications for managing heart failure (HF), coronary artery disease (CAD), and chronic kidney disease (CKD) has grown significantly, reflecting the broader recognition of their cardiometabolic benefits. In particular, SGLT2 inhibitors (SGLT2i) have evolved into guideline-directed medical therapy for HF with both a reduced and preserved ejection fraction, regardless of diabetes status, as demonstrated by event-driven trials such as EMPEROR-Preserved, EMPEROR-Reduced, DELIVER, and DAPA-HF [3,4,5,6,7,8,9,10]. Meta-analyses and the 2023 guidelines of the European Society of Cardiology (ESC) further reinforce this trend by designating a Class I recommendation for SGLT2i and GLP-1 receptor agonists (GLP-1RAs) in patients with established atherosclerotic cardiovascular disease (ASCVD) and type 2 diabetes, irrespective of their degree of glycemic control [21,22,23].

Furthermore, according to meta-analyses, the 2023 guidelines of the European Society of Cardiology for the management of CVD have established a Class I recommendation for the use of SGLT2i and GLP-1RAs in patients with established ASCVD and T2DM, irrespective of glycemic control or the use of other anti-diabetic medications [21,22,23]. The SELECT trial further expanded the role of anti-diabetic medications in cardiometabolic care. This event-driven trial demonstrated that semaglutide reduces hard cardiovascular endpoints, including cardiovascular mortality, in overweight or obese patients with ASCVD [27]. As a result, the 2024 guidelines of the European Society of Cardiology for the management of Chronic Coronary Syndromes established a Class IIa recommendation for the use of GLP-1RAs in patients with overweight or obesity and ASCVD in the absence of diabetes [82].

A prespecified secondary analysis of the SELECT trial showed that semaglutide also significantly reduced cardiovascular mortality and HF hospitalizations in patients with HF [28]. Additionally, a pooled analysis of the STEP-HFpEF program, the FLOW trial, and the SELECT trial for patients with HFpEF revealed that semaglutide significantly reduces the composite of cardiovascular death or HF hospitalization [30].

For CKD, SGLT2 inhibitors have been incorporated into the guidelines based on the results of the EMPA-Kidney and DAPA-CKD trials, irrespective of diabetes status [11,12]. Furthermore, the FLOW trial demonstrated that semaglutide improves both renal and cardiovascular hard endpoints in patients with diabetic nephropathy, regardless of body mass index [29].

Furthermore, the recently published SUMMIT trial evaluated the effects of tirzepatide on patients with HFpEF and obesity. In this double-blind, placebo-controlled study, 731 participants with an ejection fraction of at least 50% and a body mass index (BMI) of 30 or higher were randomized to receive either tirzepatide (up to 15 mg weekly) or a placebo for a minimum of 52 weeks. Over a median follow-up of 104 weeks, the primary composite outcome of cardiovascular death or worsening heart failure occurred in 9.9% of the tirzepatide group compared to 15.3% in the placebo group (hazard ratio [HR], 0.62; 95% confidence interval [CI], 0.41 to 0.95; *p* = 0.026). These findings suggest that tirzepatide may reduce the risk of cardiovascular events in patients with HFpEF and obesity [83].

Recent advancements in the treatment of MASLD highlight the potential role of GLP-1RAs and dual GLP-1/GIP receptor agonists like tirzepatide [35,36,37]. In a placebo-controlled phase II trial, semaglutide taken at 0.4 mg daily significantly improved the resolution of NASH without worsening fibrosis (59% vs. 17%; *p* < 0.001) in patients at the F2–F3 elastography stage, although it did not demonstrate a statistically significant improvement in the fibrosis stage [35]. Furthermore, semaglutide at 2.4 mg weekly in NASH-related compensated cirrhosis showed improvements in liver steatosis and cardiometabolic parameters but failed to achieve significant histologic improvements in fibrosis or NASH resolution [36]. Moreover, a phase II trial evaluating tirzepatide for MASH with moderate-to-severe fibrosis (F2–F3) at higher doses (10–15 mg weekly) achieved a resolution of MASH in 56–62% of participants compared with 10% in the placebo group (*p* < 0.001) and an improvement in fibrosis in 51% compared with 30% in the placebo group [37]. Definite conclusions regarding the efficacy of semaglutide in the management of MASLD with significant fibrosis will be provided by the ongoing phase III ESSENCE trial [84].

The efficacy of tirzepatide has also been tested in OSA, which is commonly associated with obesity and other CRHM diseases [38]. SURMOUNT-OSA trials have demonstrated the efficacy of tirzepatide in managing moderate-to-severe OSA among individuals with obesity. In two phase 3, randomized, double-blind, placebo-controlled trials, tirzepatide significantly reduced the apnea–hypopnea index (AHI), which is a key marker of OSA severity, compared with a placebo. In participants not receiving positive airway pressure (PAP) therapy (Trial 1), tirzepatide reduced AHI by 25.3 events per hour at 52 weeks compared to a 5.3 event reduction in the placebo group, yielding a treatment difference of −20.0 events per hour (95% CI, −25.8 to −14.2; *p* < 0.001). In participants already on PAP therapy (Trial 2), tirzepatide reduced AHI by 29.3 events per hour compared to a 5.5 event reduction with a placebo, with a treatment difference of −23.8 events per hour (95% CI, −29.6 to −17.9; *p* < 0.001). Secondary endpoints also favored tirzepatide, with reductions in body weight, hypoxic burden, hsCRP levels, and systolic blood pressure, along with improved patient-reported sleep quality and disturbance outcomes. These findings suggest that tirzepatide offers a novel, multifaceted approach to managing OSA in patients with obesity, targeting both respiratory events and underlying cardiometabolic risk factors [38].

The above studies highlight the expanding role of cardiologists, nephrologists, and gastroenterologist–hepatologists in prescribing anti-diabetic therapies. However, these specialists may sometimes hesitate to initiate certain treatments, particularly GLP-1RAs, potentially missing opportunities to improve clinical outcomes. Data from 2015 to 2020 showed that less than 5–10% of patients with T2DM and CVD received GLP-1RAs and/or SGLT2i despite the fact that strong evidence from trials was already available [85]. Moreover, the introduction of these therapies often requires careful reconciliation with existing glucose-lowering regimens. For instance, dipeptidyl peptidase-4 inhibitors (DPP4is) should not be combined with GLP-1RAs. In patients with HF, thiazolidinediones (TZDs) should be discontinued due to the risk of fluid retention. In patients with CKD, many glucose-lowering medications require dosage adjustments; for example, metformin is contraindicated in individuals with an eGFR <30 mL/min due to the risk of lactic acidosis [86]. Given these complexities, a specialist with comprehensive expertise in managing coronary artery disease (CAD), HF, and T2DM is critical to optimizing medical therapy and improving patient outcomes.

Although these novel medications for managing CRHM diseases present a promising path forward, several practical and systemic barriers must be addressed to ensure the broad adoption of these therapies. One major issue is cost and accessibility. Medications such as GLP-1RAs, SGLT2i, and newer dual-agonist agents can be expensive, which may limit their use in health systems with inadequate funding or among uninsured and underinsured patients. Even when these medications are recommended in guidelines, heterogeneity in reimbursement policies and coverage can further restrict their availability. Adverse effects and real-world safety also merit consideration. Clinical trials generally indicate positive safety profiles; however, there is limited evidence of long-term safety, which is being constantly evaluated. Initial concerns regarding non-ischemic anterior optic neuropathy were overestimated, and a very recent study has shown that semaglutide does not increase the risk compared to other anti-diabetic medications [87].

Many healthcare systems maintain long-standing departmental boundaries, which can impede cross-disciplinary collaboration. The lack of standardized training in integrated cardiometabolic care, combined with concerns about overlapping clinical domains, can create both logistical and professional conflicts. Without clear pathways for shared governance and consensus on role delineation, efforts to establish and operationalize a new sub-specialty may face significant institutional hurdles.

The above underscores the absence of a unified approach to managing CRHM diseases, leading to fragmented care and suboptimal outcomes. Consequently, the management of major cardiovascular risk factors—T2DM, arterial hypertension, dyslipidemia, and obesity—often remains disconnected. Addressing this challenge requires the establishment of a dedicated sub-specialty that integrates expertise across multiple disciplines. Such an integrated approach would ensure comprehensive and cohesive care, ultimately improving outcomes for the growing population of patients with CRHM diseases.

## 5. Third Pillar: Hormonal Therapies for CRHM Diseases

### 5.1. HF and Multiple Hormonal Deficiency Syndrome

Neurohormonal activation is the major pathophysiological mechanism driving HF with reduced ejection fraction (HFrEF). This model involves the excessive activation of sympathetic and renin–angiotensin–aldosterone systems and has been the cornerstone for the development of most guideline-directed medical therapies (GDMTs) for HFrEF [88]. Furthermore, metabolic abnormalities have also been implicated in the pathophysiology of both HFpEF and HFrEF, with SGLT2i forming part of the GDMT and GLP-1RA showing promising results, as already analyzed [89].

**Figure 4:**
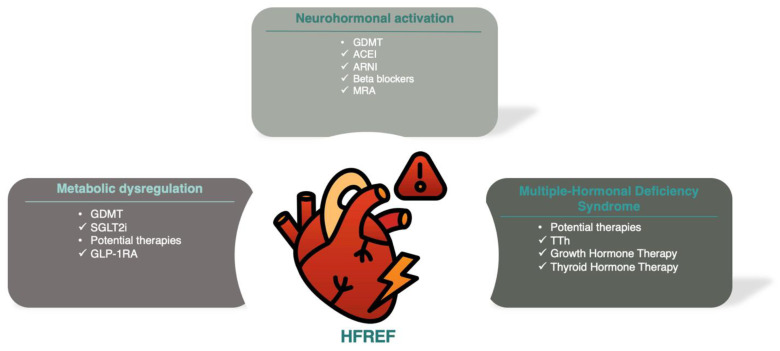
Schematic illustration of the main pathophysiologic pathways implicated in HFrEF progression and prognosis. This figure illustrates key mechanisms and therapeutic targets in HFrEF, including neurohormonal activation, metabolic dysregulation, and multiple-hormonal deficiency syndrome. Modified and used with permission from Theodorakis et al. (2025) [47].

The TOSCA registry revealed that over 90% of HF patients were deficient in at least one anabolic hormone (testosterone, dehydroepiandrosterone sulfate, insulin-like growth factor-I (IGF-I), or triiodothyronine), and more than two-thirds had deficiencies in two or more. The prevalence of IGF-I deficiency, based on age-adjusted cutoffs, was nearly 50%. More importantly, multiple hormonal and metabolic deficiency syndrome (MHDS) was associated with a significantly higher risk of hospitalization or death [42]. While these findings underscore the potential clinical impact of hormonal imbalances in HF, TOSCA is an observational study and, thus, cannot establish causality or exclude confounding factors. A schematic illustration of the main pathophysiological pathways (neurohormonal, metabolic, and MHDS) implicated in HF is presented in Figure 4. While the findings from the TOSCA registry highlight the prevalence and prognostic significance of hormonal deficiencies in HF, they are not sufficient to conclude that correcting these deficiencies will improve cardiovascular function, symptoms, or outcomes. To address this, studies have explored the potential benefits of hormone replacement therapies in this population.

Coupled with insights from MHDS in HF prognosis, TTh appears to be a promising management strategy for HF. In a systematic review of randomized controlled trials, TTh led to significant improvements in muscle strength and aerobic capacity, as well as increases in lean muscle mass and decreases in fat mass in certain trials. Additionally, improvements in insulin sensitivity and shortening of the QT interval have been reported. TTh did not consistently affect blood pressure, lipid profiles, or heart rate, nor did it lead to any serious adverse effects [43]. Another systematic review and meta-analysis of randomized controlled trials showed that, in patients with hypogonadism and HF, TTh significantly delayed the time to ischemia in patients with chronic angina, increased VO2, ameliorated heart rate, and improved insulin sensitivity. However, in this study, these benefits did not translate to improvements in the NYHA class or LVEF [90]. Furthermore, an additional meta-analysis of randomized controlled trials demonstrated that TTh significantly improved VO2 by 2.70 mL/kg/min (95% CI, 2.68–2.72 mL/kg/min) and exercise capacity, as assessed via the 6 min walk test (+54.0 m; 95% CI, 43.0–65.0 m) or the incremental shuttle walk test (+46.7 m; 95% CI, 12.6–80.9 m) [91]. It should be noted, however, that caution is advised when using TTh in patients with severe cardiac, hepatic, or renal dysfunction due to the risk of congestion and/or HF decompensation. Furthermore, as there is a lack of event-driven trials, no universal recommendation can be made for the use of TTh in patients with HF.

In addition to testosterone, growth hormones (GHs) play a role in regulating various physiological processes, including cardiovascular function. GH stimulates hepatic IGF-I secretion, which promotes protein synthesis, tissue repair, and cellular growth [44,45]. Notably, there are various reports in the literature on the local expression of GH receptors in cardiac and vascular cells, signifying potential IGF-I-independent effects of GH in the cardiovascular system. Furthermore, the local production of IGF-I by cardiac and vascular cells has been implicated in various physiological and pathophysiological processes. In the cardiovascular system, GH and IGF-I can increase myocardial mass and contractility, enhancing ventricular performance. They also reduce peripheral vascular resistance and improve endothelial function. Key non-cardiac effects include stimulating lipolysis, increasing skeletal muscle mass and strength, enhancing bone density, and exerting insulin-like glycemic effects through IGF-I [44,45]. According to our recently published meta-analysis of randomized-controlled trials, GH therapy significantly improved left ventricular ejection fraction (3.34%, 95% CI: 1.09% to 5.59%, *p* = 0.0037), peak oxygen consumption (2.84 mL/kg/min, 95% CI: 1.32 mL/kg/min to 4.36 mL/kg/min, *p* = 0.0002), and New York Heart Association class (−0.44, 95% CI: −0.08 to −0.81, *p* = 0.023). GH therapy also reduced the composite of death, worsening HF, or ventricular tachycardia by 41% (95% CI: 0.39–0.90, *p* = 0.013). Subgroup analyses indicated that patients with ischemic cardiomyopathy, baseline ejection fraction ≥30%, and longer treatment duration experienced greater benefits [45]. However, before a guideline-directed recommendation can be safely made, there is a need to conduct large-scale, event-driven, randomized-controlled trials to confirm these benefits.

In addition to TTh and GH therapy, there are additional insights for the use of T3 in the management of MHDS with low T3 syndrome. According to a meta-analysis in patients with HF and low T3 syndrome, thyroid hormone therapy significantly enhanced left ventricular ejection fraction (5.61%, 95% CI: 4.38% to 6.85%, *p* < 0.01) and cardiac output (0.65 L/min, 95% CI: 0.42 to 0.89 L/min, *p* < 0.01). Additionally, early-to-late diastolic transmitral flow velocity was higher in the thyroid hormone group compared to the control group (0.29, 95% CI 0.15 to 0.42, *p* < 0.01). Furthermore, brain natriuretic peptide levels were lower in patients receiving thyroid hormone therapy (−1.49, 95% CI: −2.15 to −0.84; *p* < 0.01) [46].

Moreover, as demonstrated in our recently published state-of-the-art review, there is emerging evidence for the use of ghrelin for HF management. Clinical trials investigating ghrelin’s acute effects in HF patients have demonstrated significant improvements in cardiac output, ranging from 15 to 30%. Moreover, one study showed that a 3-week course of ghrelin therapy significantly increased maximal oxygen consumption, lean body mass, and grip strength in HF patients. Preclinical studies further support these clinical findings, highlighting additional benefits of ghrelin, including the modulation of the autonomic nervous system, the promotion of vasodilation, the enhancement of endothelial function, the prevention of myocardial remodeling, the reduction in arrhythmogenic risk, and increased muscle mass in HF models. As a result, ghrelin is a promising therapeutic option for HF, particularly as an inotropic agent with multifaceted benefits, including autonomic nervous system modulation, anabolic effects, and metabolic regulation. However, further trials are required to confirm its long-term efficacy and safety and assess whether its benefits can translate into reductions in hard clinical endpoints [47].

Adipokines play a pivotal role in the pathophysiology of HF, with distinct implications in HFpEF and HFrEF [92]. Chronic hyperleptinemia, a hallmark of obesity, has been strongly implicated in the development of HFpEF. Elevated leptin levels in HFpEF patients are largely attributable to higher body mass index, as obesity is both a driver of HFpEF pathogenesis and a key factor in increased leptin production. Leptin contributes to insulin resistance, chronic inflammation, diastolic dysfunction, myocardial remodeling, and arterial stiffness—processes central to HFpEF. Notably, leptin levels in HFpEF are not significantly correlated with N-terminal prohormones of the brain natriuretic peptide (NT-proBNP), suggesting that leptin’s role may be more causative, reflecting systemic metabolic dysfunction rather than cardiac wall stress. This aligns with the concept of HFpEF as a metabolic-driven disease rather than a primarily hemodynamic syndrome. In HFrEF, the metabolic profile differs markedly. Despite the catabolic state often seen in HFrEF, leptin levels are significantly higher than in controls. This may be driven by neurohormonal activation, chronic inflammation, tissue hypoxia, and ectopic leptin production via hypoxia-inducible factor 1-alpha (HIF-1α) pathways. Interestingly, higher leptin levels in HFrEF are inversely correlated with NT-proBNP and associated with a lower risk of adverse outcomes, although this significance disappears after adjusting for NT-proBNP. This relationship may reflect the “obesity paradox”, where higher leptin levels in non-cachectic patients are indicative of better nutritional status and prognosis. Adiponectin, in contrast, exhibits beneficial effects on cardiovascular health and insulin sensitivity. Adiponectin levels are elevated in HFrEF, correlating positively with NT-proBNP. This elevation is likely a consequence of the catabolic state, with reduced fat stores driving increased adiponectin production [92]. Higher adiponectin levels in HFrEF are associated with a numerically increased risk of adverse events, though this association is not statistically significant after adjustment for NT-proBNP, suggesting that adiponectin reflects disease severity rather than directly influencing outcomes. However, HFpEF patients often have adiponectin levels comparable to non-HF controls, likely due to the obesity-associated suppression of adiponectin. These findings underscore the complex and contrasting roles of adipokines in HF sub-types, particularly highlighting HFpEF as a metabolic-driven condition. Therefore, future research into adipokines is crucial to further advance hormonal insights into the pathophysiology and management of HF [92].

The aforementioned studies have demonstrated the implications of hormonal dysregulation in HF pathophysiology and prognosis, as well as the potential of hormonal therapy to improve cardiac function, symptoms, and possibly hard endpoints. Despite this preliminary evidence from mechanistic studies, small trials, and meta-analyses on the use of hormonal therapies for managing MHDS associated with HF, there remains a lack of large-scale, event-driven randomized controlled trials. This limitation prevents the establishment of robust guideline-directed recommendations. Such trials are essential and should be conducted in the future to address this gap in evidence. A cardiologist, while highly skilled in advanced heart failure management, may lack the specialized knowledge required for hormonal therapies. Conversely, an endocrinologist or metabolic medicine physician may have deep expertise in hormonal regulation, diabetes, or lipid disorders but a limited understanding of heart failure pathophysiology. This gap underscores the necessity of this new sub-specialty—Cardiovascular–Endocrine–Metabolic Medicine—that integrates expertise from these fields to effectively address this emerging therapeutic domain.

### 5.2. Male FHH

Male FHH is commonly observed in conditions such as obesity, insulin resistance, T2DM, dyslipidemia, and arterial hypertension, with a bidirectional association between these conditions and low testosterone levels. TTh has been shown to improve body composition by increasing muscle mass and decreasing fat mass, as well as enhancing metabolic parameters in several studies [48]. There has been a long-running debate about the potential risks and benefits of TTh [93]. Beyond improving symptoms of hypogonadism, TTh has shown potential benefits in patients with FHH, particularly in cardiometabolic conditions [40,94]. The T4DM trial demonstrated that TTh in prediabetic men with total testosterone levels <400 ng/dL reduced the progression to T2DM by 40% over two years while also improving lean mass and sexual symptoms [40]. Furthermore, studies, including meta-analyses, have shown that TTh in adult patients with hypogonadism can improve all-cause mortality [95,96]. A generally reassuring safety profile was demonstrated in the TRAVERSE study [41]. TRAVERSE enrolled men aged 45 to 80 with symptoms compatible with hypogonadism, serum testosterone levels of <300 ng/dL, and those who were at high risk for cardiovascular events. No adverse effects of TTh were observed in blood pressure or lipid profiles, and there was no excess of major adverse cardiac events observed between the TTh and placebo groups over a mean of 22 months (hazard ratio, 0.96; 95% confidence interval, 0.78 to 1.17; *p* < 0.001 for noninferiority). However, a higher incidence of pulmonary embolism (0.9% vs. 0.5%), nonfatal arrhythmias requiring intervention (5.2% vs. 3.3%), atrial fibrillation (3.5% vs. 2.4%), and acute kidney injury (2.3% vs. 1.5%) was observed with TTh versus the placebo [41].

Combined with the generally positive effects of TTh on sexual function, quality of life, bone mass, and hemoglobin levels, its administration in patients with CRHM diseases should be considered in clinical practice, provided that there are no contraindications [48].

### 5.3. Estrogen Deficiency: Premature Ovarian Insufficiency

POI, defined as the loss of normal, predictable ovarian activity before the age of 40 years, is a cause of infertility and endocrine dysfunction in women [97]. POI, which affects approximately 1% of women under the age of 40, has adverse implications for cardiovascular health within a wider range of physical and emotional issues [98,99]. This heterogeneous disorder is characterized biochemically by amenorrhea with hypoestrogenemia and elevated gonadotrophins [98]. Spontaneous and iatrogenic POI is associated with increased risks of hyperlipidemia, hypertension, and T2DM, compared with the average age for menopause [100]. The risk of CVD is increased in women with POI [101]. While menopausal hormone therapy (MHT) can mitigate some aspects of POI, many uncertainties remain regarding optimal management; individualized care that includes attention to cardiovascular risk profiles is recommended [102].

## 6. Proposed Training Curriculum in Cardiovascular–Endocrine–Metabolic Medicine

The ongoing discussions concerning Preventive Cardiology and Cardiometabolic Medicine as potential dedicated sub-specialties or training fellowships have raised discussions about which clinicians are best suited to fill this role. These sub-specialties have been proposed as extensions of general cardiology, endocrinology, nephrology, internal medicine, or family medicine.

In our view, as reflected in the name of the proposed sub-specialty of Cardiovascular–Endocrine–Metabolic Medicine, the most suitable physicians for entering training are cardiologists, endocrinologists, or internist–diabetologists. Clinicians in these specialties could undergo a 24-month sub-specialty training program. This reflects the fact that the core of the proposed training focuses on either clinical cardiology and prevention or endocrinology, diabetes, and metabolism. Physicians already trained in these areas possess foundational expertise, making this program ideal for expanding their knowledge to include skills from other specialties and related disciplines, ensuring comprehensive care and research. Alternatively, physicians trained in internal medicine, nephrology, hepatology, chemical pathology, or vascular medicine could pursue sub-specialty training in Cardiovascular–Endocrine–Metabolic Medicine through an extended 36-month program. This would provide sufficient exposure to both parent specialties—cardiology and endocrinology—preparing them for this multidisciplinary field.

Vascular medicine, distinct from vascular surgery, focuses on the medical management of arterial, venous, and lymphatic diseases through non-invasive diagnostics, anticoagulation, and prevention. While recognized in the U.S. with ABVM fellowships, it remains uncommon globally, often integrated into cardiology, internal medicine, or vascular surgery without dedicated sub-specialization.

Chemical pathology (clinical biochemistry) varies significantly between countries. In the UK, it combines clinical and laboratory roles, focusing on metabolic disorders and diabetes mellitus with direct patient care. In Greece, known as biopathology, it is entirely laboratory-based, including microbiology, and lacks clinical involvement, rendering Greek biopathologists ineligible for the proposed sub-specialty of Cardiovascular–Endocrine–Metabolic Medicine. These differences emphasize the need for country-specific training adaptations.

**Figure 5:**
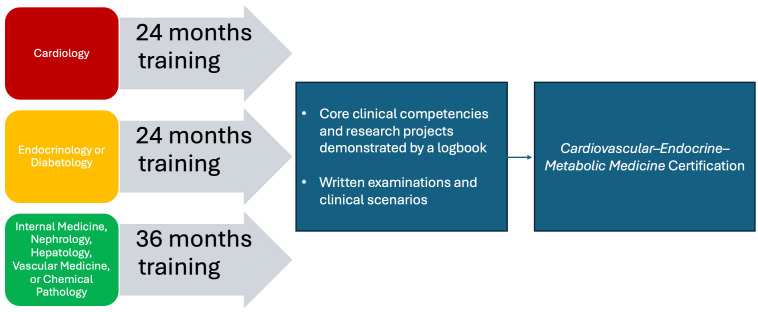
Entry pathways and duration of training for certification in the sub-specialty of Cardiovascular–Endocrine–Metabolic Medicine.

The proposed entry pathways to Cardiovascular–Endocrine–Metabolic Medicine are illustrated in Figure 5.

We hope that global societies will endorse and support the development of this sub-specialty. Organizations such as the European Society of Cardiology (ESC), the American Heart Association (AHA), the American College of Cardiology (ACC), the European Association of Preventive Cardiology (EAPC), and the American Society of Preventive Cardiology could take the lead, collaborating with the European Society of Endocrinology, the Endocrine Society, the European Association for the Study of Diabetes (EASD), the American Diabetes Association (ADA), the European Association for the Study of Obesity (EASO), and the World Obesity Federation. The involvement of other relevant specialist societies, including those in vascular medicine, nephrology, hepatology, andrology, gynecology, and chemical pathology/clinical biochemistry, could further strengthen the training framework and foster a multidisciplinary approach to ensure the success of this initiative.

The monthly distribution of training in Cardiovascular–Endocrine–Metabolic Medicine across different disciplines, based on the entry specialty, is presented in Table 1. The duration of the curriculum was founded on the idea that Cardiovascular–Endocrine–Metabolic Medicine is grounded mostly on key knowledge and expertise acquired from cardiology and endocrinology/diabetology since the diagnosis, management, and prevention of CRHM risk factors and complications are majorly covered by these two specialties. Hence, these specialists do not need dual trailing again in their core specialty. Furthermore, the disciplines of nephrology and hepatology are mostly founded on the management of early-stage CKD with medications that cardiologists and nephrologists are already familiar with, while hepatology only focuses on MASLD in the general scope of assessing and managing obesity and metabolic disorders. The reason for the relatively short rotation proposed for these two sub-specialties is because of the focused scope. On the other hand, other specialists, including nephrologists or hepatologists, have minor exposure to cardiology and endocrinology and need dual extensive training of at least 12 months in each specialty to be competitive.

The monthly distribution of training in Cardiovascular–Endocrine–Metabolic Medicine, as shown in Table 1, is based on the principle that this sub-specialty fundamentally builds upon core competencies in cardiology and endocrinology/diabetology. Specialists already board-certified in these two fields typically possess extensive expertise in diagnosing and managing the majority of CRHM risk factors and complications and, therefore, require only 24 months to gain cross-training in these complementary disciplines. By contrast, nephrologists, hepatologists, and other internal medicine sub-specialists generally have less direct exposure to the broad scope of cardiology and endocrinology. Consequently, to become fully conversant with all aspects of CRHM care, they need a longer, 36-month pathway featuring 12 months of cardiology and 12 months of endocrinology training. The shorter (5-month) rotations in nephrology and hepatology reflect the focused scope of early-stage CKD and MASLD management within this sub-specialty. Early-stage CKD is frequently encountered by cardiologists, endocrinologists, and internists, who are already well versed in reno-protective medications and interventions aimed at preventing renal function decline. Overall, this proposed curriculum ensures that each pathway addresses the specific “competency gaps” for physicians entering from different backgrounds, ultimately optimizing patient-centered, multidisciplinary care.

Below, we outline the core competencies and methods for each one of these disciplines.

### 6.1. Cardiology and Vascular Medicine

■ **Core Competencies**
  ○ Cardiovascular risk stratification.
  ○ Etiology, diagnosis, prevention, and management of arterial hypertension, including resistant hypertension, gestational hypertension, and pre-eclampsia.
  ○ Etiology, diagnosis, prevention, and management of dyslipidemia, including genetic lipid disorders and LDL apheresis.
  ○ Etiology, pathophysiology, prevention, and diagnosis of ASCVD.
  ○ Etiology, pathophysiology, diagnosis, prevention, and management of cardiometabolic HFpEF.
  ○ The basics of interpreting ECG, transthoracic echocardiography, arterial and venous color Doppler studies, coronary computed tomography, functional ischemic tests, and coronary angiography.
  ○ Emerging hormonal therapies in HF.
  ○ Research and leadership.
■ **Exclusions**: Anything outside the above, including but not limited to HFrEF, cardiac amyloidosis, cardiac transplantation, the management of ASCVD, interventional cardiology, arrhythmias, electrophysiology, and cardio-oncology, remain outside our scope.
■ **Clinical Training**
  ○ Preventive Cardiology of outpatient clinics.
  ○ HF outpatient clinics.
  ○ Echocardiography labs.
  ○ General cardiology wards.

### 6.2. Endocrinology

■ **Core Competencies**
  ○ Etiology, pathophysiology, diagnosis, prevention, and medical management of obesity, including indications for bariatric surgery.
  ○ Etiology, pathophysiology, diagnosis, prevention, and management of diabetes mellitus (including oral agents, GLP-1RAs, GIP/GLP-1RAs, insulin therapy, acute and chronic complications, and gestational diabetes).
  ○ Investigation and medical management of endocrine causes behind secondary arterial hypertension.
  ○ Key considerations for the management of thyroid disorders in patients with CVD.
  ○ Etiology, diagnosis, and management of male FHH, POI, and PCOS.
  ○ Research and leadership.
■ **Exclusions**: Anything outside the above, including but not limited to complex endocrine disorders (e.g., pituitary, thyroid, parathyroid, adrenal glands), disorders of bone metabolism, and hereditary metabolic disorders, remain outside our scope.
■ **Clinical Training**
  ○ Obesity outpatient clinics, including rotations in follow-up post-bariatric surgery.
  ○ Diabetes mellitus outpatient clinics (type 1 diabetes, type 2 diabetes, and diabetes in pregnancy).
  ○ Male and female reproduction outpatient clinics.

### 6.3. Nephrology

■ **Core Competencies**
  ○ Etiology, diagnosis, prevention, and medical management of CKD stages I to III.
  ○ Investigation and medical management of renal and renovascular secondary hypertension.
■ **Exclusions**: Anything outside the above, including but not limited to stage IV or V CKD, renal replacement therapy, renal transplantation, or glomerular diseases, remains outside our scope.
■ **Clinical Training**
  ○ CKD outpatient clinics.

### 6.4. Hepatology

■ **Core Competencies**
  ○ Etiology, pathophysiology, diagnosis, prevention, and management of MASLD.
■ **Exclusions**: Topics, including but not limited to autoimmune hepatitis, viral hepatitis, liver cirrhosis, and renal transplantation, remain outside our scope.
■ **Clinical Training**
  ○ Hepatology outpatient clinics.

### 6.5. Sleep Medicine

■ **Core Competencies**
  ○ Etiology, pathophysiology, prevention diagnosis, and basics of the management of OSA in the context of obesity.
■ **Exclusions**: Competencies beyond those listed above, including advanced management of OSA, remain out of our scope.
■ **Clinical Training**
  ○ Polysomnography laboratory.

### 6.6. Lifestyle Medicine

■ **Core Competencies**
  ○ Clinical nutrition;
  ○ Exercise prescription;
  ○ Smoking–alcohol cessation;
  ○ Behavioral counseling, including motivational interviewing.
■ **Clinical Training**
  ○ Nutrition–dietetic outpatient clinics, including in-patient consultations.
  ○ Familiarity with quantitative exercise physiology laboratory assessments, e.g., measurement of VO2 max.
  ○ Smoking–alcohol cessation and behavioral counseling clinics.

The practical implementation of training in this sub-specialty could begin with the establishment of integrated outpatient clinics that combine cardiology, endocrinology, and metabolic medicine in a single framework. For example, a specialized center might offer same-day consultations with a cardiologist, endocrinologist, and dietitian to jointly evaluate patients with obesity and/or T2DM. This model reduces the need for multiple referrals, avoids duplicative testing, and accelerates the initiation or adjustment of new cardiometabolic therapies. Another effective strategy involves creating a shared inpatient service for individuals admitted with acute decompensated HF and concomitant metabolic disorders, as well as those presenting with diabetic complications and established CVD. Here, a multidisciplinary team can address both the immediate clinical issues and long-term prevention strategies, providing an excellent environment for fellows to learn collaborative care. Rotating individuals through cardiology, endocrinology, nephrology, and hepatology departments can further expose them to the diverse facets of cardiovascular, renal, hepatic, and metabolic medicine, ultimately enriching both patient care and research. This approach trains future specialists to recognize the intricate interplay among these organ systems, equipping them to deliver truly integrated, comprehensive care.

Future research in this field should prioritize large, event-driven, randomized controlled trials that evaluate GLP-1Ras and dual agonists across the entire cardiometabolic spectrum, addressing gaps that persist in conditions like MASLD, non-diabetic CKD, PCOS, and HF or CAD without obesity. Additionally, large trials examining newer anti-obesity medications—particularly triple agonists and agents that modulate adipokine profiles—are also warranted, as mechanistic benefits have yet to fully translate into routine clinical practice. In addition, the absence of event-driven evidence for hormonal therapies (e.g., testosterone, GH, triiodothyronine, and ghrelin) in HF remains a critical area for investigation, given the preliminary promise that these treatments hold for improving outcomes. Beyond pharmacotherapies, research should also explore cost-effectiveness, patient-reported outcomes, and the real-world impact of integrated care models and multidisciplinary training curricula. Such evidence would not only optimize clinical decision-making but also strengthen the foundation for future guidelines in Cardiovascular–Endocrine–Metabolic Medicine.

## 7. Conclusions

We have introduced the concept of Cardiovascular–Endocrine–Metabolic Medicine as a potentially unifying framework to address the growing complexity of overlapping diseases such as obesity, T2DM, hypertension, dyslipidemia, hyperuricemia, early-stage CKD, MASLD, OSA, and PCOS. By integrating established specialties—such as cardiology, endocrinology, and metabolic medicine—this approach could streamline patient care, foster multidisciplinary collaboration, and promote evidence-based interventions that target the shared mechanisms underlying cardiometabolic disorders. Furthermore, a structured curriculum and well-defined core competencies have the potential to enhance both clinical practice and research, ultimately improving patient outcomes and satisfaction.

Nonetheless, it is important to acknowledge that Cardiovascular–Endocrine–Metabolic Medicine remains a developing field. Prospective long-term outcome data, pilot programs, and broader consensus among specialists are still needed to validate its feasibility and impact on real-world practice. Ongoing dialog and collaboration among cardiologists, endocrinologists, nephrologists, hepatologists, lifestyle medicine experts, and other relevant professionals will be critical for refining training standards, establishing robust protocols, and driving future innovations. In this sense, the proposed sub-specialty offers a promising vision rather than an immediate, one-size-fits-all solution—one with an evolution that will rely on continued research, shared clinical insights, and its systematic integration into existing healthcare structures.

## Figures and Tables

**Table 1:** The monthly distribution of training in Cardiovascular–Endocrine–Metabolic Medicine across different disciplines based on the entry specialty.

Training Areas	Cardiology Entry Path (24 Months)	Endocrinology or Diabetology Entry Path (24 Months)	Internal Medicine, Vascular Medicine, Nephrology, Hepatology, or Chemical Pathology Entry Path (36 Months)
Cardiology	N/A	12 months	12 months
Endocrinology	12 months	N/A	12 months
Nephrology and Hepatology	5 months	5 months	5 months
Lifestyle Medicine	6 months	6 months	6 months
Sleep Medicine	1 month	1 month	1 month

N/A, Not Applicable.

## Data Availability

No new data were created or analyzed in this study.

## Abbreviations

The following abbreviations are used in this manuscript:

ACC	American College of Cardiology
ADA	American Diabetes Association
AHI	Apnea–hypopnea index
AHA	American Heart Association
ASCVD	Atherosclerotic cardiovascular disease
ASMR	Age-standardized mortality rate
BMD	Bone mineral density
BMI	Body mass index
BP	Blood pressure
CAD	Coronary artery disease
CVD	Cardiovascular disease
CKD	Chronic kidney disease
CRHM	Cardiovascular–Renal–Hepatic–Metabolic syndrome
DASH	Dietary approaches to stop hypertension
DPP4i	Dipeptidyl peptidase-4 inhibitors
EAPC	European Association of Preventive Cardiology
EASD	European Association for the Study of Diabetes
EASO	European Association for the Study of Obesity
ECG	Electrocardiogram
ESC	European Society of Cardiology
FHH	Functional hypogonadotropic hypogonadism
GDMT	Guideline-directed medical therapy
GIP	Glucose-dependent insulinotropic polypeptide
GLP-1RA	Glucagon-like peptide-1 receptor agonist
HbA1c	Glycated hemoglobin
HF	Heart failure
HFpEF	Heart failure with preserved ejection fraction
HFrEF	Heart failure with reduced ejection fraction
HIF-1α	Hypoxia-inducible factor 1-alpha
HR	Hazard ratio
IGF-I	Insulin-like growth factor-I
LDL-C	Low-density lipoprotein cholesterol
MASLD	Metabolic dysfunction-associated steatotic liver disease
MHT	Menopausal hormone therapy
MHDS	Multiple hormonal and metabolic deficiency syndrome
NAFLD	Non-alcoholic fatty liver disease
NASH	Non-alcoholic steatohepatitis
NT-proBNP	N-terminal prohormone of brain natriuretic peptide
OSA	Obstructive sleep apnea
PAD	Peripheral artery disease
PCOS	Polycystic Ovary Syndrome
PCSK9	Proprotein convertase subtilisin/kexin type 9
PAP	Positive airway pressure
POI	Primary Ovarian Insufficiency
RCT	Randomized controlled trial
RMR	Resting metabolic rate
SGLT2i	Sodium-glucose cotransporter-2 inhibitors
siRNA	Small interfering RNA
T2DM	Type 2 diabetes mellitus
TTh	Testosterone therapy
TZD	Thiazolidinedione
VO2 max	Maximum oxygen consumption
